# Circadian Rhythm Disruption in Cancer Survivors: From Oncogenesis to Quality of Life

**DOI:** 10.1002/cam4.70353

**Published:** 2024-10-27

**Authors:** Claire O. Kisamore, Caleb A. Kisamore, William H. Walker

**Affiliations:** ^1^ Department of Neuroscience, Rockefeller Neuroscience Institute West Virginia University Morgantown West Virginia USA; ^2^ West Virginia University Cancer Institute Morgantown West Virginia USA

**Keywords:** cancer, chronotherapy, circadian rhythms, jetlag, shift work, sleep

## Abstract

**Background:**

Circadian rhythms are approximately 24‐hour cycles in physiological and behavioral processes. They are entrained to the external solar day via blue wavelength light. Disruptions in these intrinsic rhythms can lead to circadian dysfunction, which has several negative implications on human health, including cancer development and progression.

**Aims:**

Here we review the molecular mechanisms of circadian disruption and their impact on tumor development and progression, discuss the interplay between circadian dysfunction and cancer in basic scientific studies and clinical data, and propose the potential clinical implications of these data that may be used to improve patient outcomes and reduce cost of treatment.

**Materials & Methods:**

Using scientific literature databases, relevant studies were analyzed to draw overarching conclusions of the relationship between circadian rhythm dysruption and cancer.

**Conclusions:**

Circadian disruption can be mediated by a number of environmental factors such as exposure to light at night, shift work, jetlag, and social jetlag which drive oncogenesis. Tumor growth and progression, as well as treatment, can lead to long‐term alterations in circadian rhythms that negatively affect quality of life in cancer survivors.

## 1 Introduction

**FIGURE 1:**
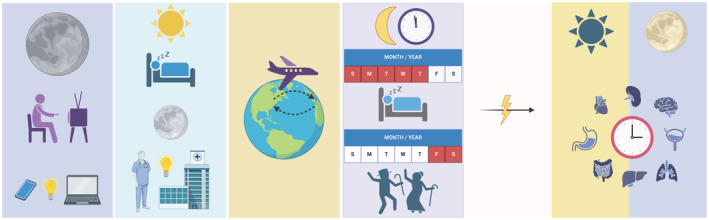
Mechanisms of circadian disruption. Light at night, shift work, chronic jetlag, and social jetlag (left to right) are prevalent mechanisms of circadian disruption. These disruptors and others lead to dampened or otherwise aberrant rhythms in physiology and behavior that hold negative consequences for health.

All living organisms possess endogenous timekeeping mechanisms that synchronize their internal cellular activity with the external environment, producing approximately 24‐h cycles known as circadian (Latin; *circa*—about, *diem*—day) rhythms. These rhythms, which are entrained by environmental cues (i.e., zeitgebers; German—time giver) such as light, temperature, food intake, and exercise regulate essential physiological processes including sleep–wake cycles [1], mood regulation [2], digestion, cellular metabolism, and immunity [3]. A growing body of evidence demonstrates the quintessential role circadian rhythms have in all facets of human health and disease. Thus, making chronobiology (the study of circadian rhythms in biological systems) a promising subject for clinically translational research initiatives [4, 5]. For example, in both clinical and pre‐clinal research, time‐restricted feeding, the consumption of food for each day within a certain window of time (i.e., 6–10 h), regulates food intake rhythms and protects against some metabolic disorders [6, 7, 8]. Randomized clinical trials establish time‐of‐day effects in antibody titers in response to vaccination [9]. Rodent studies demonstrate that drugs targeting circadian clock genes can promote wakefulness [10], reduce anxiety‐like behaviors [10], and suppress gluconeogenesis [11]. Additionally, studies indicate drugs that are already approved for widespread use can be chronomodulated (administered at a specific time of day) to reduce toxicity [12, 13, 14] or enhance outcomes such as overall survival [15, 16] as we will discuss in detail in later sections. As the body of evidence demonstrating the role of circadian rhythms in health and disease continues to grow, the fact that chronic circadian rhythm disruption (Figure 1) can contribute to the pathology of multiple diseases [2, 17] has likewise become quite clear.

Circadian rhythm disruption occurs when the body's internal clock deviates from the ~24‐h rhythm to which it is entrained and can be caused by any behavioral, physiological, or environmental factor that interferes with intrinsic rhythms. In modern society, this is typically provoked by exposure to light at night, shift work, jetlag, or social jetlag (Figure 1). Such circadian rhythm disruptors result in aberrant entrainment, leading to a cascade of downstream disruptions due to the complex, interdependent nature of the central and peripheral clocks. As the cycle breaks down, the body is unable to coordinate inter‐system communication (i.e., neuroendocrine signaling, hormonal signaling, and immune cell trafficking) which increases susceptibility to disease, further compounding dysregulated circadian rhythms.

Aberrant cellular replication, as is the defining feature of cancer, provides a unique perspective from which the bidirectional relationship between circadian rhythms and disease can be considered. For example, it is known that circadian rhythm disruption can have negative implications in cancer pathogenesis and progression [18, 19]. Additionally, many cancer survivors experience tumor‐ and treatment‐driven circadian disruption, which contributes to dysregulated sleep cycles and other related physiological abnormalities that lower the quality of life in survivors, including those in remission [20]. In this narrative review, we will discuss the molecular mechanisms of the circadian clock and its role in the cell cycle, our current understanding of the carcinogenic nature of circadian dysregulation, and current circadian biology research initiatives to improve the quality of life in cancer survivors. Lastly, we will examine practical clinical implications of our current body of knowledge on the relationship between circadian disruption and cancer.

## 2 Molecular Mechanisms

### 2.1 The Molecular Clock

The mammalian eye is a complex sensory organ that contains three classes of specialized photoreceptor cells involved in light detection. One of these classes, known as intrinsically photosensitive retinal ganglion cells (ipRGCs), is pivotal in circadian rhythm photoentrainment and unlike the other classes (rods and cones), ipRGCs are not involved in image formation [21]. Instead, ipRGCs express the G protein‐coupled receptor (GPCR) photopigment melanopsin, making them exquisitely photosensitive [22]. Melanopsin is most strongly stimulated by blue light (~480 nm), the dominant entraining zeitgeber in mammals, and responds minimally to longer red‐light wavelengths (> 600 nm) [23]. The wavelength sensitivity spectrum of melanopsin may represent an evolutionary adaptation to Earth's natural solar cycle (i.e., predictable light patterns resulting from atmospheric dispersion of solar rays leading to shorter wavelengths ~480 nm during the day with a gradual shift to longer wavelengths > 600 nm in the evening). Thus, the differential response of melanopsin to different wavelengths of solar light translates to a greater ability to discriminate between night and day, strengthening the photoentrainment of approximately 24‐h circadian rhythms [24].

Activation of melanopsin‐containing ipRGCs via blue light results in the propagation of neural impulses along ipRGC axons via the retinohypothalamic tract (RHT) to the central pacemaker (a bilaterally symmetric structure known as the suprachiasmatic nucleus [SCN] located in the anterior hypothalamus) [25, 26]. As neural impulses traversing the RHT terminate in the SCN, the excitatory neurotransmitters glutamate and pituitary adenylate cyclase‐activating peptide (PACAP) are released. Glutamate and PACAP then diffuse across the synapse and bind to receptors within the SCN core, stimulating Ca^2+^ and cyclic adenosine monophosphate (cAMP) influx and activation of intracellular signaling cascades, respectively [17, 22, 23]. Ultimately, photic information conveyed via the monosynaptic RHT to the SCN leads to increased expression of Period family genes that set the molecular clock [1]. Furthermore, peripheral clocks located in nearly all other cells of the body are set through a complex hormonal and neural crosstalk with the SCN molecular clock, ensuring 24‐h periods of systemic physiological synchronicity [27, 28].

**FIGURE 2:**
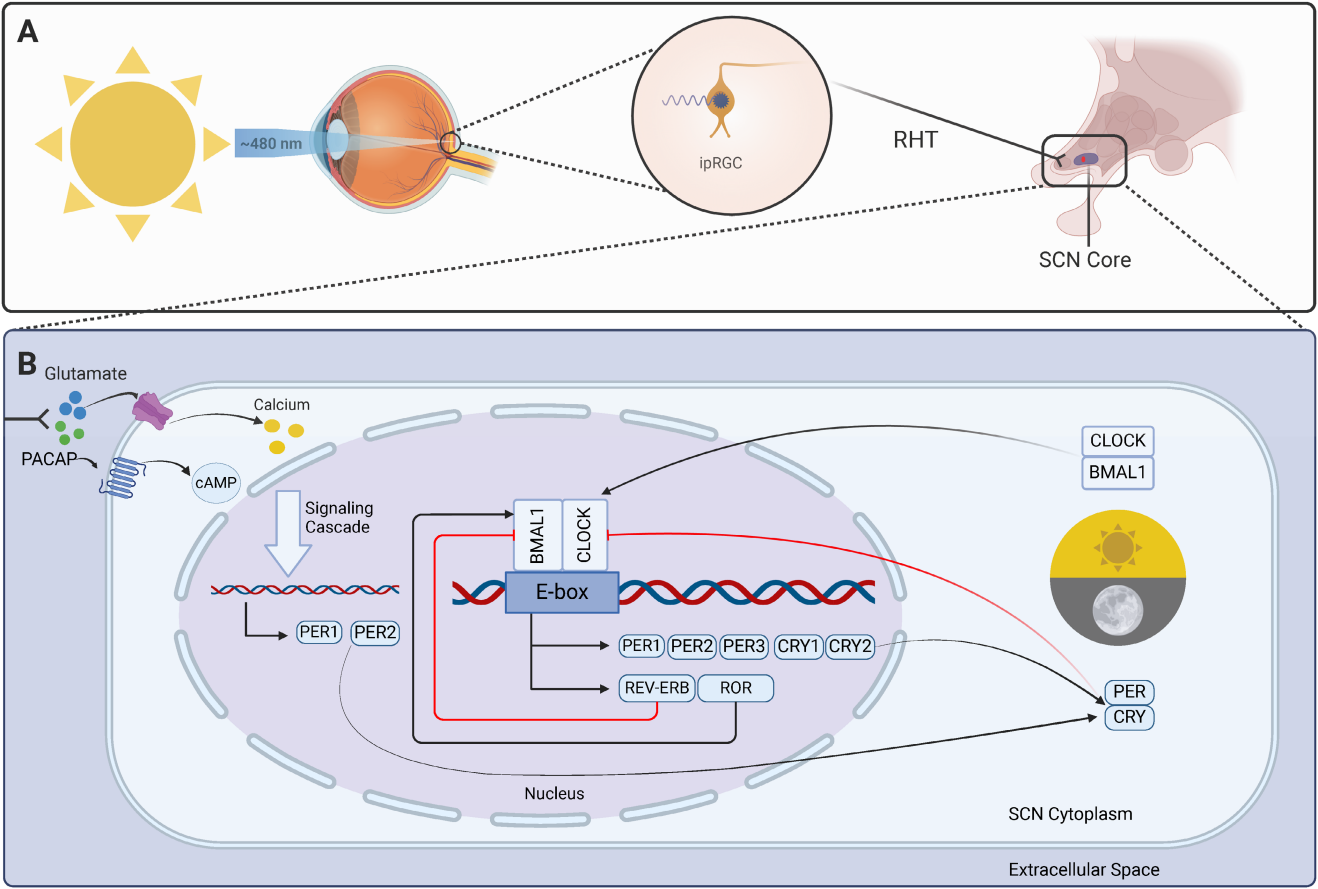
Photoentrainment of the mammalian circadian clock. (A) Blue solar light (~480 nm) enters the mammalian eye and activates melanopsin‐containing ipRGCs, resulting in the propagation of neural impulses along the RHT to SCN core in the anterior hypothalamus. (B) As these neural impulses terminate in the SCN, excitatory neurotransmitters glutamate and PACAP are released and bind to receptors on SCN cells, stimulating the production of second messengers calcium and cAMP, which trigger a signaling cascade that leads to expression of Period family genes, setting the molecular clock.

The SCN molecular clock produces rhythmic, 24‐h expression of core canonical clock components via the interaction of multiple transcriptional‐translational feedback loops [17, 26] (Figure 2). The primary feedback loop begins with the formation of circadian locomotor output cycle kaput (CLOCK) and brain and muscle ARNT‐like protein 1 (BMAL1) protein heterodimers in the cytosol of SCN cells [29]. The CLOCK‐BMAL1 heterodimeric complex translocates to the nucleus where it binds to specific DNA elements (E‐boxes) in the promoter region of period (*PER1, PER2, PER3*) and cryptochrome (*CRY1* and *CRY2*) genes, resulting in their expression [19]. PER and CRY proteins then heterodimerize, translocate to the nucleus, and repress their own transcription by acting on CLOCK‐BMAL1 complexes [30]. Investigation of this feedback loop in mice has demonstrated time‐of‐day differences in the activation of these heterodimer complexes, such that activation of CLOCK‐BMAL1 occurs in the early morning (via activation of ipRGC with blue light), leading to transcription of *Per* and *Cry* genes in the afternoon, and formation of PER‐CRY complexes that represses CLOCK‐BMAL1 transcription in the evening/night [28]. BMAL1 is further regulated by an interacting feedback loop in which the CLOCK‐BMAL1 complex activates the expression of nuclear receptors (nuclear receptor subfamily 1 group Ds [REV‐ERBs] and retinoid‐related orphan receptors [RORs]) whose protein products competitively bind ROR response elements in the BMAL1 promoter. REV‐ERBs repress the transcription of BMAL1 whereas RORs activate it. Together, these two interacting feedback loops form the basis of the central SCN molecular clock [29].

### 2.2 The Molecular Clock in Cancer

Several aspects of cell cycle progression and maintenance are regulated by core clock machinery. For example, PER1 binds Ataxia‐Telangiectasia Mutated (ATM) and checkpoint kinase 2 (CHK2) to form a complex that shields p53 from ubiquitination, allowing for cell cycle arrest and DNA repair in response to radiation‐induced double‐strand DNA breaks (e.g., UV sun rays) [31, 32]. These data and others suggest that PER1 has important tumor‐suppressing abilities [33, 34]. Indeed, attenuation of PER1 expression in murine mammary tumors leads to increased tumor cell proliferation and a shortened survival [33]. Data from human breast cancer patients also demonstrate a positive correlation between PER1 levels and overall survival [33]. Furthermore, a meta‐analysis of PER1 expression in human breast cancer survivors indicates that low levels of intratumoral PER1 are associated with poorer prognoses [33]. Outside of breast cancer, PER1 has similar implications in ovarian cancer [35], gastric cancer [36], endometrial cancer [37], head and neck squamous cell carcinoma [38], oral squamous cell carcinoma [39], and non‐small cell lung cancer (NSCLC) [40]. For example, NSCLC patient samples contained decreased levels of PER1 compared to normal lung tissue [40]. This has been attributed to hypermethylation in the PER1 promoter region [40], leading to attenuated expression.

PER2 also has strong implications in oncogenesis. It preserves p53 activity by preventing its ubiquitination via mouse double minute 2 (MDM2) [31, 41]. Mice homozygous for a mutation in *Per2* (*Per2* ^*m/m*^) demonstrate increased sensitivity to tumor development compared to their WT peers following γ radiation [42]. *Per2* ^*m/m*^ mice also lack an increase in clock gene transcription that is apparent in WT mice following irradiation [42]. Furthermore, PER2 suppresses metastasis by acting as a transcriptional corepressor of genes like twist‐related protein 1 (*TWIST1*) and snail family transcriptional repressors (*SNAI*s) that are crucial for epithelial‐to‐mesenchymal transition (EMT) [43]. In breast cancer patient samples, hypoxic conditions lead to PER2 degradation [43], supporting the general positive correlation between intratumoral PER2 and overall survival [36, 44, 45, 46, 47, 48]. Together, the evidence clearly outlines critical anti‐tumor properties of Period genes. These data provoke the postulation of Period genes as viable drug targets, specifically through stabilization of their expression. Indeed, RNA stability and abundance can be targeted through nucleic acid‐based therapeutics [49]. One could postulate that a treatment targeting *PER2* mRNA could be given alongside traditional therapies to patients with normal (i.e., non‐mutated) *PER2*. If such a treatment were to be administered, the intrinsic rhythms (i.e., time of peak Period gene expression) of the target tissue would need to be considered. Protein modulation, however, would require extra consideration due to potential post‐translational modifications.

Although there are limited data in cancer survivors, evidence suggests that clock gene disruption could play a role in disease severity (Table 1). For example, a 2021 study in elderly patients with thyroid nodules revealed aberrant expression of clock‐related proteins in normal tissue surrounding malignant nodules (> 5 mm away from the nodule) [50]. Compared to tissue from healthy patients or patients with non‐malignant nodules, CLOCK and BMAL1 levels were significantly increased in tissue surrounding malignant nodules. The opposite was true for CRYs (i.e., higher levels were discovered in non‐malignant thyroid tissue). Of note, the authors established that poor sleep quality (assessed via the Pittsburgh Sleep Quality Index [PSQI]), sleep latency, and daytime dysfunctions (e.g., mood disturbances, lack of energy, increased errors at work, and worrying about sleep) were independent risk factors for developing malignant thyroid nodules [50]. CLOCK and BMAL1 overexpression also inhibits cell growth and stalls G1 to S phase transition in human colon cancer cells [51]. Furthermore, the same study demonstrated an increased resistance to paclitaxel in human colon cancer cells overexpressing CLOCK and BMAL1. This was concluded to be due to the prevention of cell cycle progression to the G2/M phases induced by the drug [51]. However, BMAL1 overexpression increased sensitivity to oxaliplatin in a mouse model using a human colorectal carcinoma cell line. BMAL1 expression has also been correlated with higher overall survival in patients with colorectal cancer [52], nasopharyngeal carcinoma [53], metastatic melanoma [54], and others. Taken together, these studies reveal potentially protective intrinsic clock mechanisms that have uncertain effects on drug efficacy. Further studies are needed to determine the effects of not just clock proteins, but clock dimers (i.e., PERs/CRYs and BMAL1/CLOCK) on specific anti‐tumor treatments.

**TABLE 1:** The effects of core clock components on tumor development/growth.

Clock component	Species	Cancer type	Effect	References
PER1	Human (cell lines and patient samples)	Breast	Tumor suppressor; higher levels correlated with longer overall survival	[33]
	Human (cell lines)	Oral squamous cell carcinoma	Overexpression leads to suppression of glycolysis, glucose uptake, proliferation, and PI3K/AKT pathway	[34]
	Human (patient samples)	NSCLC	Decreased levels compared to healthy lung tissue; hypermethylated promoter region	[35]
PER2	Human (cell lines and patient samples)	Breast	Corepressor of EMT genes; degradation in hypoxic conditions	[38]
	Human (cell lines and patient samples)	Mixed	Higher levels correlated with longer overall survival	[39, 40, 41, 42, 43, 44]
	Mouse	Radiation‐induced lymphoma	Mutants displayed increased susceptibility to tumor development following irradiation and decreased clock gene transcription	[37]
BMAL1	Mouse	Human colorectal carcinoma	Increased sensitivity to oxaliplatin	[47]
	Human (cell lines and patient samples) and Mouse	Colorectal	Expression correlated with longer overall survival	[48]
	Human (patient samples)	Nasopharyngeal carcinoma	Expression correlated with longer overall survival	[50]
	Human (patient samples)	Metastatic melanoma	Expression correlated with longer overall survival	[51]
BMAL1/CLOCK	Human (patient samples)	Malignant thyroid nodules	Significantly higher levels in tissue surrounding malignant nodules compared to tissue surrounding non‐malignant nodules	[45]
	Human (cell lines)	Colon	Overexpression stalls G1 to S phase transition and confers resistance to Paclitaxel	[46]
CRYs	Human (patient samples)	Malignant thyroid nodules	Significantly higher levels in tissue surrounding non‐malignant nodules when compared to tissue surrounding malignant nodules	[45]

When clock genes are mutated, circadian control of the cell cycle can become dampened or lost. For example, a study conducted in Finnish patient samples (*n* = 101) concluded that *CLOCK* was mutated in 53% of colorectal cancer samples with microsatellite instabilities [55]. Single nucleotide polymorphisms (SNPs) may also play a role in an individual's risk of developing cancer. Certain SNPs in Period/Cryptochrome genes, BMAL1 and CLOCK have been reported to correlate with increased risk of cancer (reviewed extensively in [56]). However, the data on specific types of cancer and demographics of patients correlated to SNPs and increased risk of cancer is unclear. For instance, a meta‐analysis examining publications with patient samples concluded that there is not a significant correlation between CLOCK SNPs and risk of developing breast cancer [57]. Further, a systematic review examining The Cancer Genome Atlas, Cancer Therapeutics Response Portal, and The Genomics of Drug Sensitivity in Cancer databases concluded that very few samples contain mutations in core clock genes themselves. Rather, mutations in clock‐controlled genes in the cell cycle (i.e., EP300 and CREBBP) were more common [58]. Because clinical data come from patients only after they have developed cancer, it remains unclear exactly how circadian rhythm disruption alters the cell cycle on a molecular level due to the difficulty of separating the effects of core clock disruption on oncogenesis and tumor progression, and vice versa.

## 3 Circadian Disruption and Cancer

### 3.1 Circadian Disruption and Oncogenesis

One of the most common causes of circadian rhythm disruption in modern times is exposure to artificial light at night (ALAN). Since the Industrial Revolution, electric lighting has been widely implemented across the planet, causing severe light pollution in densely populated locations. Currently, around 80% of the world's population is consistently exposed to ALAN. This number increases to > 99% when only considering populations of Europe and the United States [59]. Recently, the popularity of light‐emitting diodes (LEDs) has only compounded this problem. LEDs emit primarily blue light (440–495 nm) with a wavelength similar to solar blue light that cues entrainment of the central clock (i.e., the suprachiasmatic nucleus) [60]. This becomes largely problematic after sunset, when mammals have historically stopped receiving this type of photic input. Constant blue light stimuli (e.g., phones, television, and other electronic devices) after sunset dampens the rhythm of melatonin production, ablates rhythms in Period family gene expression, and suppresses rhythmic expression of other core clock genes within the hypothalamus, liver, and other peripheral tissues [18, 61]. A recent meta‐analysis examining 19 studies internationally from 2001 to 2023 denoted a positive correlation between exposure to both indoor and outdoor sources of ALAN and the risk of developing breast cancer in women [62]. The authors also noted a lack of existing data sufficient enough to draw similar conclusions in other cancer types. Interestingly, one 2024 study analyzed satellite images obtained by the Visible Infrared Imaging Radiometer Suite (VIIRS), the Defense Meteorological Program Operational Linescan System (DMSP‐OLS), and the International Space Station (ISS). Pixel analysis of data collected by the ISS demonstrated a 1.8× increase in the instances of colorectal cancer in participants living within the highest tertile of blue light exposure [63]. However, these results varied with the data source (i.e., VIIRS vs. DMSP‐OLS vs. ISS) [63]. Similar studies using geographical ALAN exposure data and models demonstrate positive correlations between higher ALAN exposure and development of pediatric papillary cancer [64] and lung cancer [65]. However, other similar studies conclude that there is not a significant correlation between ALAN exposure and prostate cancer [66], endometrial cancer (in postmenopausal women) [67], acute lymphoblastic leukemia (in Hispanic juveniles) [68], or breast cancer [69, 70]. It is evident that there is no concrete consensus within the field, however, it appears as if the majority of studies report a significant correlation between ALAN exposure and risk of developing breast cancer in women [71]. For other cancer types, more data is needed to draw significant conclusions.

Although the most common disruptor of circadian rhythms is exposure to blue wavelength light after dark, these disturbances can also be provoked by shift work. Night shift work, defined as working outside of daylight hours (including both rotating and split shifts) [5], disrupts rhythms in core clock genes and alters melatonin secretion. This in turn dampens estrogen and aromatase‐inhibiting properties of melatonin [5], leading to an increased susceptibility to reproductive cancers. In fact, suppression of rhythms in melatonin secretion is so crucial that The International Agency for Research on Cancer and the National Toxicology Program (United States) have classified night shift work as a potential carcinogen [5]. Epidemiological studies have also revealed an increased risk of breast cancer in women who work night shifts [72, 73, 74]. Indeed, a study spanning 10 years analyzed 562 female night shift nurses and reported a 23% increase in risk of breast cancer in premenopausal women who worked at least 3 days of night shift per month for 1–14 years compared to non‐night shift workers. This risk increased to 30% in postmenopausal women working for ≥ 30 years [75]. Other studies have also reported an increased risk of developing breast cancer in female night shift workers [73, 74, 76], however, data from these studies overwhelmingly report only a modest increase in risk. These results have been attributed to increased exposure to light at night, which disrupts secretion of pineal melatonin [77]. Another potential contributor to developing breast cancer could be SNPs. A 2014 French study conducted in night shift nurses revealed that SNPs rs1482057 and rs12914272 in *RORA* were correlated with breast cancer in these women [78]. Within the group of women with breast cancer who had one of these SNPs, there was also a significant correlation with the rs11932595 mutation in *CLOCK* [78]. Shift work has also been correlated with an increased risk of developing endometrial [79], colorectal [72], and prostate [80, 81] cancer. Additional studies fail to present a significant correlation between extended night shift work and increased risk of developing breast [77, 82, 83] or ovarian [84, 85, 86] cancer. Likely, many other factors such as duration of night shift work [73, 75, 76, 87], disrupted sleep cycles [73], inappropriate timing of food intake [18], and genetics [88] contribute to the risk of developing breast and other cancers in night shift workers.

Another type of circadian disruption, jetlag, can be split into two categories: chronic jetlag (CJL) and social jetlag (SJL). Although limited to a very small population of people, CJL is a common model of general circadian disruption in animal research. This is typically accomplished by a phase shift (advancement or delay of intrinsic rhythms) in animals via the light cycle (i.e., lights come on 6–8 h earlier every few days) [89, 90, 91]. In mice, circadian dysfunction via CJL drives oncogenesis and metastasis of hepatocellular carcinoma [90], Lewis lung carcinoma [92], pancreatic ductal carcinoma [93], radiation‐induced lymphoma [94], and breast cancer [95]. Murine models allow for testing mechanistic hypotheses like disruption of rhythms in pineal melatonin [96], elevated stemness/ability to metastasize [95, 97], and increased inflammation [98]. However, the ways in which CJL affects cancer development in humans is less understood. This is potentially due to the very specific population of people affected by CJL (i.e., pilots and flight attendants who regularly fly internationally). In flight personnel, a 2008 meta‐analysis spanning 13 years and 21 studies reports a 70% increased risk of developing breast cancer and a 40% increased risk of developing prostate cancer [99]. Additional retrospective studies spanning 60 [100], 50 [101], and 42 [102] years also demonstrate that airline pilots (most of whom routinely flew international routes) have an increased risk of developing prostate cancer [100, 101] and skin cancers (melanoma, non‐melanoma, and basal cell carcinoma) [100, 101, 102]. This risk increases with exposure to cosmic radiation and hours flown [100, 101, 102]. The findings of a self‐reported survey of participants of the Harvard Flight Attendant Health Study suggest that female flight attendants also have an increased risk of developing both melanoma and non‐melanoma skin cancers as well as breast cancer [103]. However, it is unclear if the increased risks presented in these studies are due exclusively to circadian dysfunction. It is likely that other environmental factors (i.e., radiation exposure) also provoke oncogenesis of airline pilots and flight attendants. Due to these confounds and the very limited pool of subjects (i.e., airline pilots and flight attendants) that are affected by CJL, this form of circadian disruption is not fully understood in humans. However, foundational studies in animal models clearly illustrate a connection between CJL and cancer risk [90, 94, 104] and progression [92, 93, 104, 105, 106] that is independent of cancer type.

While CJL is limited to a small population, SJL is experienced much more prevalently. Social jetlag refers to the modern tendency to wake up earlier and sleep for shorter durations on weekdays while “catching up” on sleep during weekends [107] and has increasingly been the subject of studies by circadian biologists. Approximately 50% of the current work force/students experience ≥ 2 h of SJL, and around 70% experience ≥ 1 h [108]. Particularly of interest in the modern medical landscape is how SJL impacts public health (reviewed in [109]). At least 1 h of habitual SJL has been identified as a potential contributing factor in prostate [110] and lung [111] cancer. An interesting confound of these findings comes from a 2006 study demonstrating that people who lived lifestyles involving chronic SJL were more likely to engage in behaviors that increase the risk of cancer like drinking alcohol and smoking tobacco [107]. Studies on the mechanisms of how SJL potentially contributes to or otherwise affects oncogenesis are incredibly sparse and therefore represent a largely untapped area of research potential. While it is often difficult to separate the effects of circadian disruption on oncogenesis from resulting circadian disruption following oncogenesis, the data discussed thus far suggest that initial circadian disruption plays at least a moderate role in tumor development. This should therefore be considered by physicians when evaluating an individual's risk of developing cancer.

### 3.2 Circadian Disruption in Cancer Survivors

So far, we have considered cancer as a consequence of circadian dysregulation, however, the reverse relationship (i.e., circadian dysregulation as a consequence of cancer) is of equal clinical relevance. In cancer survivors, aberrant rhythms in hormones like melatonin and cortisol indicate circadian disruption. Indeed, breast cancer survivors have lower levels of nighttime melatonin [112] as well as lower amplitudes of rhythms in melatonin [112]. This aberrant melatonin expression is likely responsible for the poor sleep quality observed in these patients [112, 113]. In fact, supplementation of treatment with melatonin has been linked to better sleep quality, improved drug efficacy, and even a longer overall survival [114, 115]. Not only does this help to normalize rhythms and lead to better sleep quality, but melatonin has anti‐tumor and immunomodulatory functions [116]. Interestingly, one study demonstrated that supplementary treatment with melatonin helped to normalize aberrant rhythms in cortisol in patients with advanced‐stage solid tumors [117]. Rhythms in cortisol release, which typically peak in the morning, can be dampened in lung [118], ovarian [119], and breast [120] cancer. In patients with metastatic breast cancer [121], renal cell carcinoma [122], lung cancer [123], and epithelial ovarian cancer [124], cortisol rhythms have been used to predict survival, with flatter rhythms indicating poorer prognoses. Flattened cortisol rhythms may be indicative of increased stress, which is also correlated with decreased quality of life [125]. Indeed, nighttime salivary cortisol levels have been negatively correlated with quality of life in patients with head and neck cancers [125]. A study including 30 women who were 5+ year survivors of ovarian cancer concluded that dampened cortisol rhythms were associated with higher fatigue and lower quality of life [126]. Similar results have been demonstrated in breast cancer survivors [127].

Other than dampened expression of important rhythmic hormones, consequences of general circadian disruption are prevalent in cancer survivors. Even in remission, issues like chronic fatigue and decreased sleep quality lower the quality of life in survivors [128, 129]. One study used wearable actigraphs to measure circadian parameters in early‐stage breast cancer survivors (156 adult women who underwent surgical treatment followed by chemotherapy) 1 year after receiving their last chemotherapy treatment. The mesor (24 h rhythm‐adjusted activity counts), amplitude (distance between peak and trough activity), peak activity, and circadian quotient (strength of the rhythm) of survivors were consistently 5%–15% lower than those of healthy controls [130]. This indicates that breast cancer survivors may have dampened/disrupted activity rhythms compared to their peers. Studies in metastatic colorectal cancer [131], and various late‐stage cancers (including colorectal, pancreatic, lung, and liver) demonstrate similar results [132]. Furthermore, fatigue and weight loss, which are typically indicators of chemotherapy‐induced circadian disruption [133], are correlated with a decreased median survival rate in metastatic colorectal cancer [133, 134] and have significant associations with circadian disruption in breast [135], pancreatic [132], lung [132], liver [132], and esophageal [136] cancer. As discussed above, disruptions in these rhythms aggravate oncogenic mechanisms, meaning that continued disruptions could lead to prolonged disease. Broadly, it is possible that tumor‐driven circadian disruptions instigate further tumor progression, in turn potentially decreasing the overall rate of remission in survivors.

Sleep disturbance represents a particularly harmful morbidity affecting the quality of life of cancer survivors. In fact, a meta‐analysis of 160 clinical studies assessing symptom prevalence in 46,279 cancer survivors found that 60.7% experience significant sleep disturbance [137]. Sleep quality is a multidimensional concept, however, and management strategies to restore the quality of sleep require an understanding of the complex underlying etiology. A small study of 20 women diagnosed with stage I‐III breast cancer who had not yet started chemotherapy determined that 71% of them had difficulty sleeping (via PSQI), and more than half had sleep efficiencies lower than 85% [138]. Similar findings were reported in a meta‐analysis of 27 studies involving cancer survivors which revealed an average pre‐treatment PSQI of 7.11 (where > 5 indicates “poor sleep”) [139]. Studies directly assessing the treatment‐independent effects of cancer on sleep should be expanded, however, to account for the psychological confound of coping with a recent cancer diagnosis. For this, animal models are extremely useful, as psychological stress is a much less significant variable. Research also demonstrates the chronic effects of the administration of cytotoxic chemotherapeutics on circadian rhythms and sleep, complicating the search for an underlying etiology. For example, a study examining 180 women undergoing various treatment regimens for breast cancer demonstrated a 10% decrease in sleep efficiency after their first round of chemotherapy compared to their baselines [140]. After the last round of chemotherapy, sleep efficiency was only 5% lower than their baselines [140]. Altogether, these results suggest that both a tumor‐driven mechanism (i.e., independent from treatment) and a treatment‐dependent mechanism contribute to circadian disruption and sleep disturbance in cancer survivors.

For survivors of childhood cancer, the effects of administering high doses of toxic treatments during crucial developmental periods are largely unknown. One study, however, reported that exposure to high amounts of anthracycline before the age of 21 was correlated with decreased sleep quality in adulthood [141]. It also examined metrics of sleep outcome including PSQI scores, insomnia, and nightly sleep duration [141]. Survivors of pediatric cancer (diagnosed before the age of 21 and have since completed treatment at least 5 years prior to the study) were compared to a random sample of closest‐aged siblings of the survivors. Cancer survivors were more likely to report an overall sleep duration of less than 6 h as well as a longer latency to fall asleep. PSQI scores of survivors were higher (indicating poorer sleep quality) than those of siblings, and survivors also reported higher frequencies of awakenings and sleep medication use [141]. These data indicate that survivors of pediatric cancers suffer long‐term effects on sleep, however, it is unclear if these effects are caused by circadian rhythm disruption from the cancers themselves, or if an underlying disruption contributing to oncogenesis still exists into adulthood. It is also worth noting that the two‐process model of sleep, which states that two independent mechanisms drive sleep regulation (the sleep/wake homeostatic process and the circadian pacemaker process) [142], poses the possibility that mechanisms driving sleep disruption in cancer survivors are independent of circadian disruption. It is more likely, however, that circadian disruption before, during, or after tumorigenesis and treatment in large part contributes to prolonged sleep disturbances. Like adults, children report fatigue (not to be confused with sleepiness) as one of the most common treatment‐related side effects [143]. The National Comprehensive Cancer Network (NCCN) defines cancer‐related fatigue (CRF) as a distressing lack of energy that cannot be improved with normal amounts of rest or sleep. This definition encompasses physical, mental, and emotional exhaustion. CRF is intermingled with sleep disorders in cancer survivors, causing issues such as insomnia, excessive daytime napping, and increased sleep latency (i.e., the time it takes to fall asleep) that decrease quality of life [143]. Indeed, a study conducted in adults who had acute lymphoblastic leukemia during childhood determined that the survivors performed lower on an executive function test than a control population. This was correlated with increased fatigue and decreased processing speed and attention in female survivors [144]. Given the data discussed thus far, it is plausible to attribute these long‐term symptoms in survivors of childhood cancer in part to circadian disruption experienced before, during, and after cancer treatment. Overall, evidence indicates an effect of circadian rhythm disruption on cancer outcomes, ranging from mild alterations in sleep patterns and alterations in melatonin, cortisol, and activity rhythms to predictors of overall/median survival.

## 4 Clinical Research Challenges and the Future of Chronotherapy

Despite tremendous leaps in our understanding of molecular oncology and expansion of the therapeutic arsenal at our disposal, the human and financial cost of cancer today remains staggeringly high. Cancer currently ranks as a leading cause of death in every country of the world [145], responsible for an estimated 10 million deaths in 2020 [146]. A decision analytical modeling study in 2017 estimated the global financial cost of cancer from 2020 to 2040 to equal $25.2 trillion [147]. The astronomical cost of cancer globally demonstrates the need to expand upon current therapeutic algorithms to incorporate more patient‐specific factors in treatment regimens. Such a personalized approach to medicine, though not without its challenges, is becoming more feasible as technology and our understanding of cancer at the molecular level evolves [148, 149, 150].

A practical, promising, and rapidly evolving field of study within the realm of personalized medicine includes the discipline of chronotherapeutics. There are three components to the practice of chronotherapy: “training the clock,” “drugging the clock,” and “clocking the drug” [151]. “Training the clock” involves promoting circadian hygiene in patients (i.e., going to bed/waking at the same time each day, time‐restricted feeding, exercise, etc.) [152] to improve disease outcome. The term “drugging the clock” refers to therapeutics that modulate core clock proteins [153]. The last approach, termed “clocking the drug,” will be the primary focus of chronotherapeutic discussion in this review. This approach broadly seeks to optimize existing therapies via individualizing the time of administration of a given therapy to maximize treatment efficacy while minimizing deleterious side effects [154]. This practice takes advantage of an individual patient's chronotype, a term which describes the general time of day at which a person prefers to be awake and productive. Colloquially, this is described as either a “morning lark” or a “night owl.” [155]. Circadian rhythmicity, as previously described, is a process that occurs in all nucleated cells, is coordinated by the central pacemaker (i.e., the SCN), and results in a predictable phenotypic pattern of cellular activity that ultimately determines how a patient will respond to a given therapy. Chronotherapeutic modulation is particularly well suited to therapies with a narrow therapeutic index as it allows for a more conservative treatment schedule, thus reducing exposure to potentially harmful levels of a therapy. Chronotherapeutic optimization of cancer therapies, termed cancer chronotherapy, is perhaps the most well studied application of this principle.

Cancer chronotherapy was first introduced to clinical practice in the early 1970's by endocrinologist Franz Halberg [156]. His pioneering work was described in a case study published in 2006 which detailed the successful treatment of a then 21‐year‐old female that had presented with a rare and highly malignant (10% 2‐year survival rate) ovarian endodermal sinus tumor requiring 20 successive courses of chronomodulated chemotherapy [157]. Since Halberg's conception of cancer chronotherapy, a tremendous effort has been made within the scientific community to understand the basic biological underpinning of circadian‐based medicine, ultimately culminating in the 2017 Nobel prize in physiology and medicine being awarded to Jeffrey C. Hall, Michael Rosbash, and Michael W. Young for their work in elucidating the molecular mechanism of the circadian clock. This recognition of the utility of circadian‐based therapy has helped usher into the clinic a renewed interest in discovering the extent to which chronotherapy can improve patient outcomes, specifically resulting in an increase in the number of randomized clinical trials focusing on chronomodulated therapy schedules.

For example, several Phase III clinical trials comparing conventional time‐independent treatment schedules to chronotherapy schedules have demonstrated improved tolerability by up to fivefold and double the efficacy of the treatment [158]. A systematic review of 16 randomized controlled trials (RCTs) evaluating chrono‐chemotherapy and chrono‐radiotherapy in the treatment of head and neck cancer also found statistically significant toxicity reduction while maintaining treatment efficacy [159]. Additionally, a retrospective study examining 97 patients with NSCLC that had metastasized to the brain found that stereotactic radiosurgery (SRS) administered in the morning (10 AM‐12:30 PM; *n* = 59) experienced significantly improved 3‐month local control, overall survival, and less CNS‐related deaths [160]. These studies and others on various anatomic sites (hematologic, head and neck, lung, colorectal, cervical, breast, and brain cancers—reviewed extensively in [161]) highlight both the growing momentum that circadian‐based medicine has generated since the 2017 Nobel prize and clearly describes the significant impact that chronotherapy can have in treating patients with cancer. Furthermore, in this review, the authors identify among many other notable findings three critical takeaways from clinical trials that we will highlight here. (1) The peripheral circadian clocks among anatomically distinct cell types operate independent of one another, demonstrating the need to develop distinct chronomodulated therapy schedules for anatomically distinct cancers. A representative example of this principle includes the time‐of‐day discrepancy in normal tissue toxicity that results from radiation therapy of head and neck cancers (HNC) vs. cervical cancers, in which greater toxicity (mucositis) for HNC occurred with evening administration, while the opposite was true for cervical cancer [162, 163, 164, 165]. An additional variable that is likely playing a role in this observed effect and is certainly responsible for other discrepancies among chronomodulated therapy is patient gender, which brings us to the second critical finding. (2) Patient gender as a variable must be accounted for in future clinical trials, as a plethora of evidence exists demonstrating variability in chronotherapy response among men and women. For example, a clinical trial comparing a chronomodulated (chronoFLO4, 4‐day course of 1, fluorouracil‐leucovorin (FU‐LV) infusions from 2215 to 0945 with peak delivery at 0400 h and 2, oxaliplatin from 1015 to 2145 with peak delivery at 1600 h) and a conventional (FOLFOX2, 2‐h infusion of oxaliplatin‐LV between 0900 and 1600 h and a constant rate of infusion of FU for 22 h on day 1, followed by 2‐h infusion of LV only between 0900 and 1600 h and a constant rate of infusion of FU for 22 h on day 2) delivery schedule for the administration of chemotherapy in colorectal cancer patients found that only males experienced a survival benefit from the chronomodulated therapy, while females experienced increased survival with conventional therapy [166, 167]. (3) Finally, although many clinical trials examining chronomodulated schedules for cancer treatment have focused solely on chemotherapy or radiotherapy, immunotherapy represents another major therapeutic option that can be optimized via specific timing of administration [168]. Retrospective studies in matched cohorts of patients with melanoma [169, 170, 171], NSCLC [172, 173], and metastatic squamous cell carcinoma of the esophagus [174] have found that immune checkpoint inhibitor (ICI) medications ipilimumab (anti‐CTLA‐4), nivolumab (anti‐PD1), and pembrolizumab (anti‐PD1) demonstrated improved efficacy when administered in the morning compared to evening administration. Additionally, a recent foundational science study demonstrated a circadian rhythm in the expression of PD1 in CD8+ T cells, further supporting the efficacy of chronomodulated immunotherapies [175]. In general, the consensus of those analyzing chronotherapeutic data suggests sufficient cause to consider chronotherapy a viable treatment strategy, with the caveat that larger, multicentric RCTs with rigorous standardized protocols is necessary to maximize its impact [176, 177, 178].

The role of chronotherapy extends beyond the optimization of treatment efficacy and reduction of side effects for patients with cancer, also providing a means to control the astronomical costs of drug production. According to a report published in 2017 on clinical drug development for (1) colorectal, (2) breast, and (3) NSCLC from 1979 to 2014, mean drug development times and total attrition rates (represented here as *x*‐years to % attrition rate) between phase I and III clinical trials were (1) 6.7 years to 87%, (2) 8.9 years to 83.9%, and (3) 6.6 years to 92%, respectively [177]. Many of these drugs failed in later phases [179], meaning much of the average drug development cost of $2.6 billion USD had already been invested. Chronotherapy research on these drugs may yet salvage the capital investments and more importantly increase the yield of efficacious drugs on the market. This principle is perfectly illustrated by the real‐life example of a chemotherapeutic agent oxaliplatin that failed to progress in phase I trials due to unfavorable toxicity [180]. After development ceased, another company investigating the safety and efficacy of the drug demonstrated that chronomodulated delivery of oxaliplatin effectively ameliorated toxicity concerns and provided the greatest efficacy [181]. These findings were validated in Phase II trials and later confirmed in randomized Phase III trials, proving the concept that simply by retooling the dosing regimens to better reflect the patient's biological rhythms, chronotherapy has the potential to revive a multitude of orphan drugs known to be safe in humans [166, 182, 183, 184, 185].

The primary goal of personalized medicine is to maximize the clinical efficacy of a given therapy while minimizing its side effect profile [186]. Chronotherapy aims to accomplish this goal by incorporating the historically overlooked temporal dimension into the decision‐making calculus that informs the development of therapeutic algorithms. In this way, chronotherapy offers a unique and promising mechanism of therapy optimization, however, inconclusive evidence from select clinical trials demonstrates that such an approach is not without limitations. For example, a study investigating the role of a circadian schedule (evening administration) of methotrexate and 6‐mercaptopurine did not find a significant difference in event‐free survival (no evidence of relapse) among children with acute lymphoblastic leukemia compared to conventional morning dosing [187]. Additionally, an RCT comparing morning and evening dosing of cisplatin for patients with NSCLC found no significant difference in disease outcome [188]. These findings highlight some of the existing knowledge gaps that cancer researchers and oncologists must overcome.

Another fundamental challenge facing cancer chronotherapy is the dramatic inter‐individual variation of chronotypes and the differential response of therapies that result from this metabolic variation [178]. These variations have historically been accounted for via clinical questionnaires in which patients reported whether they considered themselves a “night owl” or “morning lark” [189]. This manner of categorizing chronotypes fails to capture the complexity of biological rhythms and provides very little support to chronotherapeutic efforts. However, with the advent of wearable devices capable of tracking a patient's real‐time circadian rhythms in high resolution [190], the future of personalized chronotherapy remains promising.

One major caveat to the practice of chronomodulated medicine is the rhythms of the tumors themselves. Loss of clock gene function weakens circadian regulation of the cell cycle (reviewed in [191]), thus potentially losing the synchronicity of tumor cells among each other. Therefore, the heterogeneous rhythmicity of these cells may result in aberrant rhythms or even complete loss of rhythmicity [192, 193]. In some cases, this affects rhythms throughout the body [194], presenting a major hurdle in the practice of chronotherapy. These complications can sometimes be relieved by using clock‐modulating drugs [195], thus combining the “drugging the clock” and “clocking the drug” approaches of chronotherapy. Despite these shortcomings, chronotherapy represents a potential paradigm shift in how clinicians treat patients with cancer. To maximally exploit the potential of cancer chronotherapy in the clinic, collaboration between basic scientists, physicians, regulatory bodies, technology developers, and pharmaceutical companies is essential. Clinical big data and meta‐analytical studies are promising solutions to the structural gaps that impede implementation of chronotherapy on a larger scale [176]. Pooling clinical data from electronic health records (EHR) into a centralized chronotherapy database that reports factors such as patient chronotype, molecular characteristics of their cancer, and time of day delivery of cancer therapy could dramatically accelerate growth in the field of chronotherapy.

## 5 Conclusions and Future Directions

In this review we have explored the molecular mechanics of circadian rhythms in humans and model organisms, explained common causes of circadian disruption, discussed the complex relationship between circadian disruption and cancer, and considered the clinical applications and challenges facing cancer chronotherapy. Together, this review provides a clinically translational perspective of the chronotherapeutic arm of cancer research and offers practical solutions like chronomodulated drug scheduling and re‐imagined clinical trials to realize the full potential of cancer chronotherapy. The convergence of advanced big data system technology with our growing understanding of cancer pathophysiology and individual chronotype variability represents a potential nexus for the emergence of personalized medicine.

**FIGURE 3:**
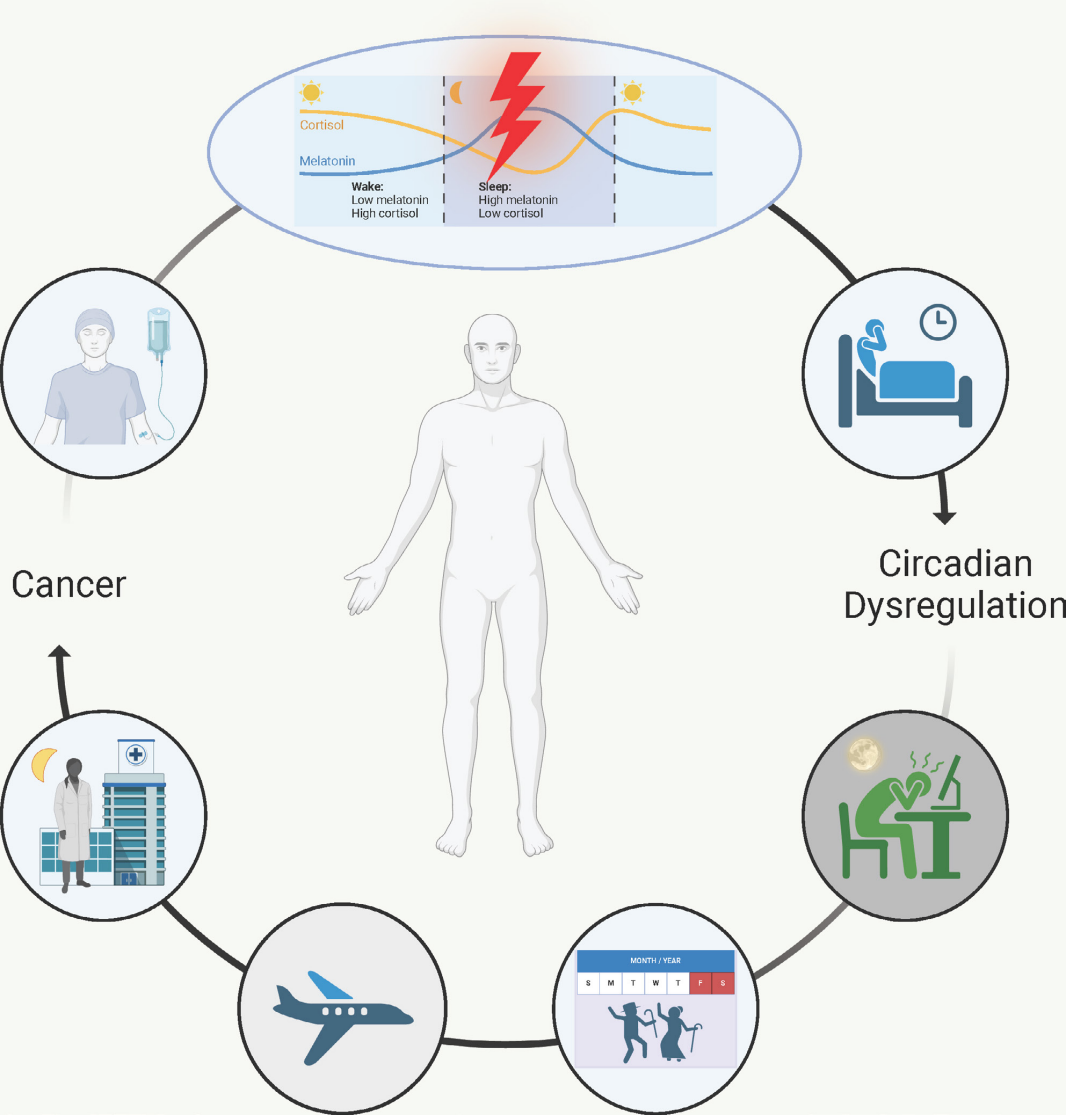
Bidirectional relationship between circadian dysregulation and cancer. Cancer is a known disruptor of melatonin and cortisol signaling which decouples the natural circadian rhythm, leading to circadian dysregulation. Furthermore, chemotherapy and insomnia are independent sequelae of cancer that likewise lead to circadian dysregulation. Artificial light at night (ALAN), social jet lag (SJL), chronic jet lag (CJL), and night‐shift work are all circadian disruptors linked to cancer.

More research is needed to understand the reciprocity between circadian disruption and tumor growth, development, and metastasis (Figure 3). Specifically, long‐term analyses of survivors are needed to determine the consequences of tumor‐ and treatment‐driven circadian dysfunction. Moving forward, clinicians should consider circadian rhythm disruption when both evaluating an individual's risk for developing cancer and prescribing anti‐tumor therapy. Chronotherapy should be considered in clinical trials of anti‐tumor drugs, especially those that do not demonstrate increased efficacy in earlier stages. Infrastructure for around‐the‐clock treatment options and chronomodulated dosing should be more widely available across cancer types. This includes implication of wearable technology that accurately assesses patient chronotypes to promote a more personalized treatment protocol. Overall, circadian rhythms and time‐of‐day effects should be carefully considered when prescribing and administering treatment.

## Author Contributions

**Claire O. Kisamore:** conceptualization (equal), writing – original draft (lead), writing – review and editing (equal). **Caleb A. Kisamore:** conceptualization (equal), writing – original draft (equal), writing – review and editing (equal). **William H. Walker II:** conceptualization (equal), funding acquisition (lead), resources (lead), supervision (lead), writing – original draft (equal), writing – review and editing (equal).

## Conflicts of Interest

The authors declare no conflicts of interest.

## Data Availability

Data sharing is not applicable to this article as no new data were created or analyzed in this study.
